# Elevated Pentraxin 3 in Obese Adipose Tissue Promotes Adipogenic Differentiation by Activating Neuropeptide Y Signaling

**DOI:** 10.3389/fimmu.2018.01790

**Published:** 2018-07-30

**Authors:** Min-Kyung Shin, Bongkun Choi, Eun-Young Kim, Ji-Eun Park, Eui Seung Hwang, Hyang Ju Lee, Min Kyung Kim, Ji-Eun Kim, Seong Who Kim, Eun-Ju Chang

**Affiliations:** ^1^Department of Biomedical Sciences, Asan Medical Center, University of Ulsan College of Medicine, Seoul, South Korea; ^2^Stem Cell Immunomodulation Research Center, Asan Medical Center, University of Ulsan College of Medicine, Seoul, South Korea; ^3^Department of Biochemistry and Molecular Biology, Asan Medical Center, University of Ulsan College of Medicine, Seoul, South Korea

**Keywords:** pentraxin3, neuropeptide Y, obesity, adipogenesis, macrophage

## Abstract

Obesity is accompanied by chronic systemic inflammation characterized by macrophage infiltration of obese tissues, an elevated plasma level of inflammatory substances, and excessive accumulation of lipids. The pro-inflammatory factor pentraxin 3 (PTX3) is also elevated in obese tissues, suggesting its potential role in adipogenesis. We found by analyzing murine preadipocyte 3T3-L1 cells, and human adipocytes derived from mesenchymal stem cells, which locally elevated PTX3 in obese adipose tissue augments adipocyte differentiation and subsequent lipid accumulation. This occurs *via* the upregulation of adipogenesis-related transcription factors. PTX3 enhanced lipid accumulation in murine 3T3-L1 cells by upregulating the expression of neuropeptide Y (NPY)/NPY receptor (NPYR) expression in preadipocytes. Pharmacological inhibition by NPYR antagonists abolished these effects. NPY also promoted the production of reactive oxygen species (ROS), a known trigger of adipogenesis. NPYR antagonists as well as antioxidant *N*-acetylcysteine showed anti-adipogenic effects by reducing the ROS levels, indicating that PTX3 mediates adipogenesis through NPY-dependent ROS production. These findings suggest that PTX3 plays a key role in the development of obesity by enhancing adipocyte differentiation and lipid synthesis *via* NPY/NPYR signaling. These observations provide a mechanistic explanation for the adipogenesis mediated by PTX3.

## Introduction

Obesity is characterized by the excessive accumulation of adipose tissue accompanied by a chronic state of inflammation and is closely associated with an increased risk of metabolic disorders including type 2 diabetes, dyslipidemia, and hypertension (1, 2). Although the major function of adipose tissue, which is primarily composed of adipocytes, is fat storage, it also plays an important role in metabolic homeostasis and energy balance by secreting a variety of bioactive molecules as an endocrine organ (3, 4). Adipose tissue expands *via* the production of new adipocytes from preadipocytes (adipogenesis) and their subsequent storage of lipid droplets (5, 6). During adipogenesis, preadipocytes undergo proliferation and differentiation into mature adipocytes, which involves a coordinated activation of transcription factors such as CCAAT/enhancer-binding protein alpha (C/EBP-α), peroxisome proliferator-activated receptor gamma (PPAR-γ), and fatty acid binding protein-4 (FABP-4) (7–9). The dysregulation of free fatty acids and inflammatory factors released by enlarged adipose tissue has been suggested to be associated with the pathogenesis of obesity-related metabolic complications (1).

Obesity-related inflammation in adipose tissues accelerates the exacerbation of metabolic dysfunction (10, 11). The change of resident adipocytes and the production of inflammatory cytokines caused by infiltrating macrophages in obese individuals are related to the aggravation of obesity-induced inflammation that leads to the pathogenesis of metabolic disease (12–14). The accumulation of macrophages in adipose tissue has been shown to be positively correlated with obesity-related inflammation (15). Increased recruitment of macrophages to adipose tissue in obesity results in the secretion of various pro-inflammatory cytokines (1, 14) that potentially contribute to the differentiation of adipocyte progenitors (3). The macrophage populations in adipose tissue are a significant determinant of obesity-related inflammation (16). Indeed, the recruitment of monocytes/macrophages into adipose tissue is associated with expansion of adipose tissue and the enlarged adipocytes observed in obese individuals (14). Moreover, the macrophage number in adipose tissue significantly correlates with body mass index and adipocyte size (17). The infiltration of adipose tissue by macrophages is accompanied by increased levels of tumor necrosis factor-α (TNF-α) and chemokine (C-C motif) ligand 2 (CCL2) (16). Moreover, several studies have reported that pathological adipose tissue expansion due to adipocyte hypertrophy (cell size increases) plays an important role in obesity development (6, 18–20). This initiates adipose tissue dysfunction and also causes the development of insulin resistance (18–20).

Pentraxin 3 (PTX3), a member of the long pentraxin family, is a pattern recognition molecule that functions in innate immunity (21) and is a useful biomarker of a pro-inflammatory status (22). PTX3 is principally produced by immune cells, including monocytes/macrophages and neutrophils. Vascular-related cells such as endothelial cells and smooth muscle cells also produce PTX3 (23) in response to inflammatory stimuli such as TNF-α, interleukin-1β (IL-1β), and lipopolysaccharides (24). In addition, Abderrahim-Ferkoune et al. have reported that PTX3 expression is induced by TNF-α in adipocytes (25). Previous studies have reported that PTX3 gene expression is higher in obese (ob/ob) and obese diabetic (db/db) mice (25) and in the adipose tissue of obese human subjects (26, 27), suggesting a possible link between PTX3 and obesity. However, the precise effects of PTX3 on obesity remain unclear. To further elucidate this in our present analysis, we focused on the potential association between PTX3 secreted from macrophages and adipogenesis. Our findings indicate that PTX3 has a significant effect on adipocyte differentiation and lipid accumulation *via* neuropeptide Y (NPY)/neuropeptide Y receptor (NPYR) signaling.

## Materials and Methods

### Reagents

Insulin, indometacin, Oil-Red-O stain, and *N*-acetylcysteine (NAC) were obtained from Sigma-Aldrich (St. Louis, MO, USA). Recombinant PTX3 protein was purchased from R&D Systems (Minneapolis, MN, USA). 3-isobutyl-1-methylxanthine (IBMX), NPY, and NPY receptor antagonists (BIBO3304, BIIE0246, and CGP71683 hydrochloride) were obtained from Tocris Bioscience (Tocris House, IO). Dexamethasone was obtained from Enzo Life Sciences (Farmingdale, NY, USA).

### Mouse Study

C57BLKS/J lar- + Lepr^db^/ + Lepr^db^ (db/db) (5-week-old, ~33 g average bodyweight) and C57BLKS/J lar- m +/m + wild-type (WT) mice (5-week-old, ~20 g average bodyweight) were purchased from SLC (Shizuoka, Japan). The animals were maintained at the Animal Center of Ulsan University with free access to food and drinking water under 12-h cycles of light and dark. For plasma PTX3 measurement, mice were anesthetized, plasma was snap-frozen in liquid nitrogen, and stored at −80°C until use. Adipose tissues were embedded in paraffin for immunohistochemical analysis. The studies with mice were conducted according to the protocol, which was granted by the Ethics Committee of Ulsan University (Seoul, Korea) and conformed to the Guide for the Care and Use of Laboratory Animals published by NIH. The application form included a statement guaranteeing strict observation to the animal’s rights.

### Cell Culture

Murine 3T3-L1 preadipocytes were grown in Dulbecco’s Modified Eagle’s Medium (DMEM, Thermo Fisher Scientific Inc., Rockford, IL, USA) supplemented with 10% fetal bovine serum (Thermo Scientific), penicillin (100 U ml^−1^, Invitrogen Life Technologies, Carlsbad, CA, USA), and streptomycin sulfate (Invitrogen Life Technologies). Adipose tissue was obtained through liposuction from the healthy volunteers (23–26 years old) provided with informed consent. Human mesenchymal stem cells (hMSCs) were isolated from human fat tissue in accordance with Institutional Review Board guidelines (IRB number 2012-0283, Asan Medical Center, Korea). The protocol was approved by the Institutional Review Board guidelines (Asan Medical Center, Korea). All subjects gave written informed consent in accordance with the Declaration of Helsinki. In brief, adipose tissue was washed with phosphate-buffered saline (PBS) and mechanically chopped before digestion with 0.2% collagenase I (Sigma) for 1 h at 37°C with intermittent shaking. The digested tissue was washed with alpha-minimum essential medium (α-MEM) containing 15% fetal bovine serum and then centrifuged at 1,000 rpm for 10 min to remove mature adipocytes. hMSCs were maintained in alpha-minimum essential medium (α-MEM) supplemented with 10% fetal bovine serum (28, 29). Cells were maintained in a humidified incubator at 5% CO_2_ and 37°C.

### RNA Isolation, RT-PCR, and quantitative real-time PCR (qPCR)

Total RNA was extracted from cultured 3T3-L1 cells and hMSCs using Trizol reagent (Invitrogen Life Technologies) following the manufacturer’s instructions. RevertAid First strand cDNA Synthesis kit (Thermo Scientific) was used to synthesize cDNA from RNA and PCR was performed in a BIO-RAD T100™-Thermal Cycler (Bio-Rad, Hercules, CA, USA). The PCR products were analyzed by electrophoresis in 2% agarose gel and imaged using a UV gel Imaging system (Bio-Rad). qPCR analysis was performed in optical 96-well plates using SYBR Green PCR master mix (Roche, Penzberg, Germany) and the Light Cycler 480 Real time-PCR Detection system (Roche) in accordance with the manufacturer’s instructions. The PCR primers used in these analyses are listed in Table S1 in Supplementary Material. Gene expression was normalized to that of GAPDH, which was used as an internal control. The relative expression of the target genes was calculated with a standard curve method using the target Ct values and the Ct value for GAPDH.

### Gene Silencing and Transfection

The transfection of small interfering RNA (siRNA) against PTX3 was performed as previously described (30). The combination of four sequences of siRNA oligonucleotides, a SMARTpool of siRNA to PTX3 (ON-TARGET plus Human PTX3), and negative control siRNA were purchased from Thermo Scientific Dharmacon (Lafayette, CO, USA). hMSC cells were transfected with siRNA using the transfection reagent RNAiMAX (Invitrogen, Carlsbad, CA, USA). Silencing efficiency was evaluated by qPCR.

### Preadipocyte Differentiation and Oil-Red-O Staining

Adipogenesis was conducted in 3T3-L1 cells as described previously (13, 31). Briefly, 3T3-L1 preadipocytes were seeded into 12-well plates at a density of 2 × 10^5^ and grown until they reached confluence (designated day 0). To induce differentiation, the growth medium was replaced with DMEM containing an adipogenic differentiation supplement: 3-isobutyl-1-methylxanthine (IBMX, 0.25 mM), dexamethasone (2 µg/ml), indometacin (0.125 mM), and insulin (5 µg/ml) (day 0). The positive-control cells were treated with insulin. To evaluate the effect of PTX3 or NPY on adipogenesis and lipid synthesis, the adipogenic differentiation supplement was removed, and the cells were then fed with DMEM containing the indicated concentrations of PTX3 or NPY for another 4 days. In some experiments, NAC (10 mM) or NPY receptor antagonists were also applied at the time of differentiation induction as follows: NPY1R antagonist (BIBO3304, 1 µM), NPY2R antagonist (BIIE0246, 1 µM), or NPY5R antagonist (CGP71683 hydrochloride, 1 µM). The medium was replaced every 2 days until day 6. Six days after the induction of differentiation, 3T3-L1 cells were washed once with PBS and fixed with paraformaldehyde (4% in phosphate buffer) for 30 min at room temperature. After washing once with distilled water, the fixed cells were incubated with 60% isopropanol for 2 min. Isopropanol was then removed and the lipid droplets in mature adipocytes were stained with Oil-Red-O solution (6:4, 0.6% Oil-Red-O dye in water) for 30 min. Finally, the cells were washed three times with distilled water and the stained lipid droplets were visualized and photographed using a microscope (Leica, Wetzlar, Germany).

### Adipogenic Differentiation of Human Mesenchymal Stem Cells

Adipogenic differentiation of hMSCs was performed by incubating the cells with adipogenesis induction medium (MEM supplemented with 2 µg/ml dexamethasone, 0.25 mM IBMX, and 5 µg/ml insulin) for 3 days, followed by 3 days of culture in maintenance medium (MEM alone) as described previously (32). Positive control cells received an additional treatment with indometacin (0.125 mM) (33). The medium was changed every 3 days until day 9. The hMSCs were treated with the indicated concentrations of PTX3 or NPY with or without NAC (10 mM) throughout the period of incubation to evaluate the effects on adipogenesis. Lipid synthesis was detected with Oil-Red-O staining. The hMSCs transfected with control- or PTX3-siRNA were incubated with adipogenesis induction medium and stained with Oil-Red-O.

### Reactive Oxygen Species (ROS) Measurement

The ROS levels were measured using a dichlorofluorescin diacetate (DCFDA) Cellular ROS Detection Assay Kit (Abcam, Cambridge, MA, USA) following the manufacturer’s instructions. Briefly, 3T3-L1 cells or hMSCs were stained with 2′, 7′-DCFDA for 30 min at 37°C and then treated with various concentrations of PTX3 or NPY in the presence or absence of NAC (10 mM) or NPY receptor antagonists. After a 4 h treatment, the cells were analyzed using a BD FACSCanto™ Flow Cytometer (BD Biosciences, San Jose, CA, USA).

### Immunohistochemistry

Serial 5-µm paraffin-embedded sections of obese tissues from WT lean and db/db obese mice were incubated with PTX3 or NPY antibodies (Abcam) and developed using the REALTM EnVisionTM Detection System Peroxidase/DAB + kit (Dako) in accordance with the manufacturer’s protocols as described previously (34). Tissues were photographed with an Olympus BX51 microscope outfitted with an Olympus DP72 digital camera (Olympus).

### PTX3 Measurements

The murine plasma PTX3 concentrations were measured using a PTX3 specific sandwich ELISA kit (R&D systems, Minneapolis, MN, USA) in accordance with the manufacturer’s protocols as described previously (35). All samples were examined in triplicate for each experiment.

### Statistical Analysis

**Figure 1:**
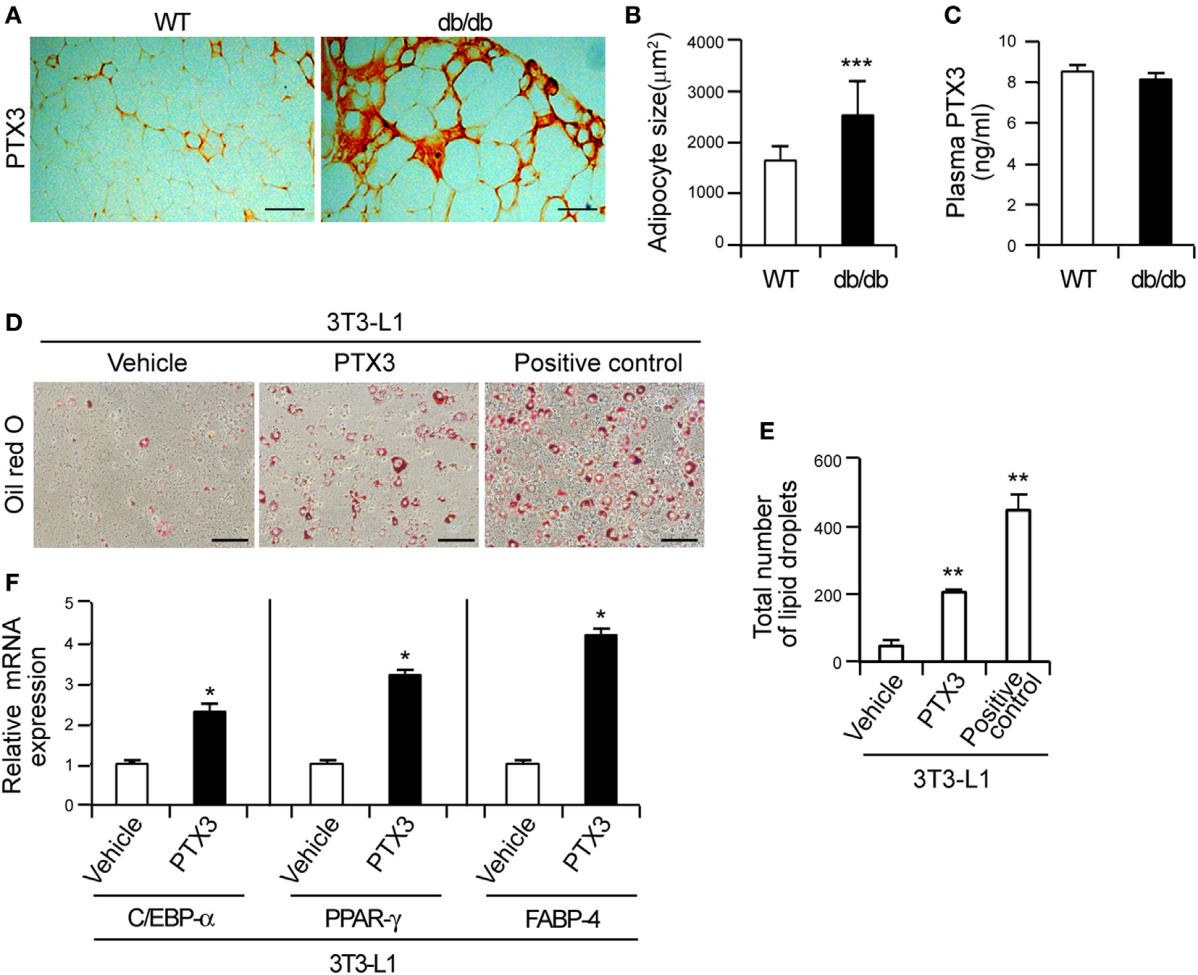
Elevated pentraxin 3 (PTX3) in obese adipose tissue induces adipocyte differentiation. **(A)** PTX3 immunohistochemical (IHC) signals in adipose tissue from wild-type (WT) and db/db mice. This IHC staining pattern demonstrates a marked increase of adipocyte cells in the db/db mouse. Scale bar: 200 µm. **(B)** The size of adipocytes of WT (*n* = 20) and db/db (*n* = 20) mice was determined from **(A)**. **(C)** Circulating PTX3 protein levels in plasma from WT (*n* = 5) and db/db (*n* = 5) mice determined by ELISA. **(D)** PTX3 promotes lipid accumulation in 3T3-L1 cells. 3T3-L1 preadipocytes were induced to differentiate into adipocytes by culturing in the presence of a differentiation cocktail for 2 days. Cells continuously treated with 5 µg/ml insulin for an additional 4 days were used as positive controls. In the PTX3-treated cells, the differential cocktail was replaced with PTX3 (200 ng/ml) and the cells were cultured for an additional 4 days without insulin. The cells were then fixed, stained with Oil-Red-O, and imaged under a microscope. Scale bar: 100 microm (µM). **(E)** Quantification of the total number of lipid droplets in **(D)** (*n* = 5). **(F)** PTX3 upregulates the expression of adipocyte differentiation markers. 3T3-L1 preadipocytes were induced to differentiate into adipocytes. C/EBP-α, peroxisome proliferator-activated receptor γ, and fatty acid binding protein-4 mRNA expression levels in the cells shown in **(D)** were determined using quantitative real-time PCR (*n* = 4). Data are representative of three independent experiments. These data are presented as the fold-changes of the mean vehicle control value. The data values represent the mean ± SD. *p < 0.05, **p < 0.005, and ***p < 0.0005 compared with the vehicle control. *p* values were calculated using Mann–Whitney test **(B,C,F)** or Kruskal–Wallis test **(E)**.

**Figure 2:**
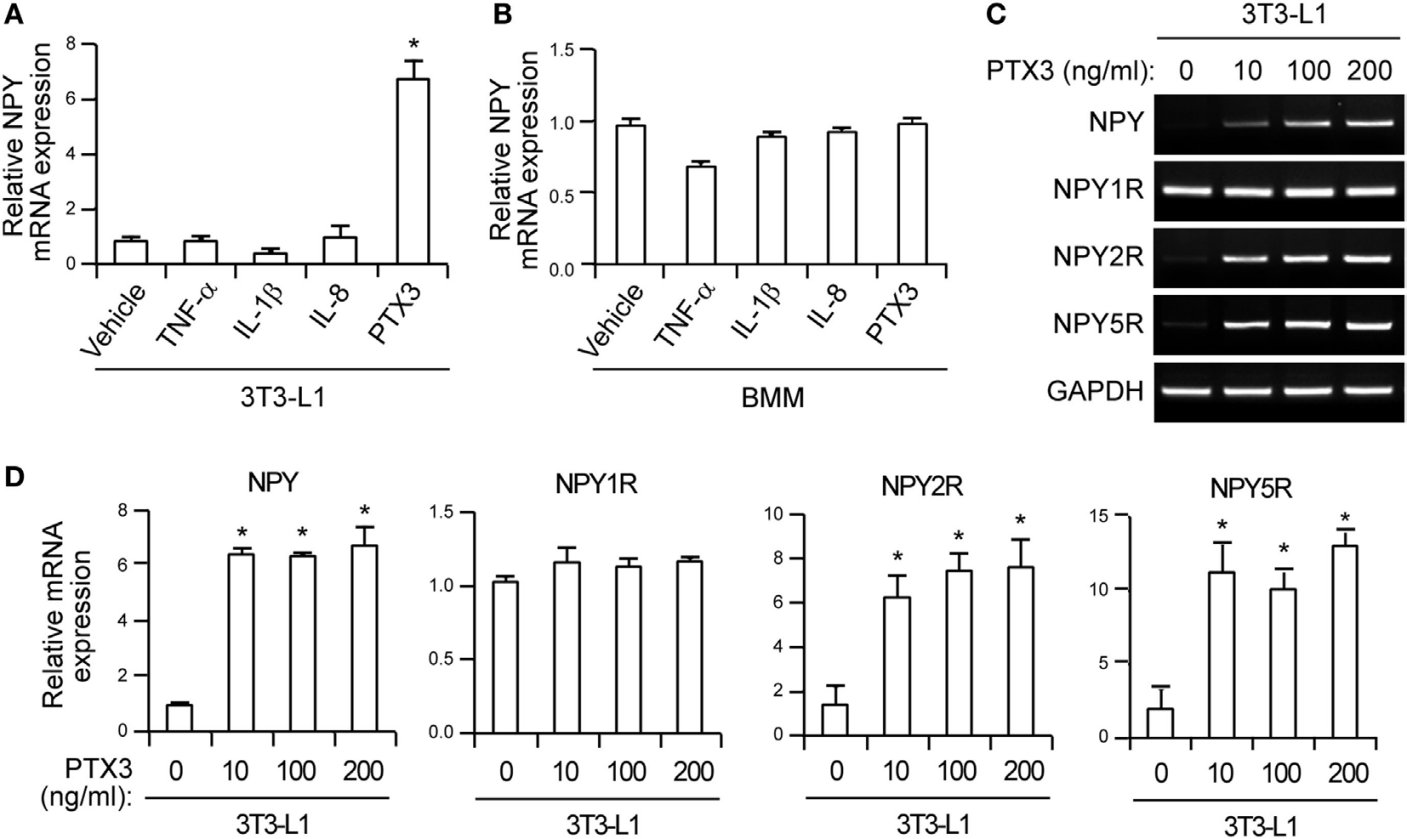
Pentraxin 3 (PTX3) induces neuropeptide Y (NPY) and neuropeptide Y receptor (NPYR) expression in murine preadipocytes. **(A,B)** 3T3-L1 cells **(A)** and bone marrow-derived macrophages **(B)** were treated with the tumor necrosis factor-α, interleukin-1β, IL-8 cytokines, and PTX3 (10 ng/ml) for 24 h. The mRNA expression level of NPY was determined using quantitative real-time PCR. GAPDH was used as an internal control (mean ± SD). Data values are the fold-changes of the mean vehicle control value (*n* = 4). **(C)** Effects of PTX3 on NPY/NPYR expression in 3T3-L1 cells. 3T3-L1 cells were incubated with the indicated concentration of PTX3 for 24 h and the mRNA expression of NPY, NPY1R, NPY2R, and NPY5R were determined using RT-PCR. GAPDH was used as an internal control. **(D)** PTX3 upregulates the expression of NPY, NPY2R, and NPY5R. The NPY, NPY1R, NPY2R, and NPY5R mRNA levels in 3T3-L1 cells treated with the indicated concentrations of PTX3 for 24 h were quantified using quantitative real-time PCR (*n* = 4). Data are representative of three independent experiments. Data are the fold-changes in the mean vehicle control value and represent the mean ± SD. *p < 0.05 compared with the vehicle control. *p* values were calculated using Kruskal–Wallis test.

**Figure 3:**
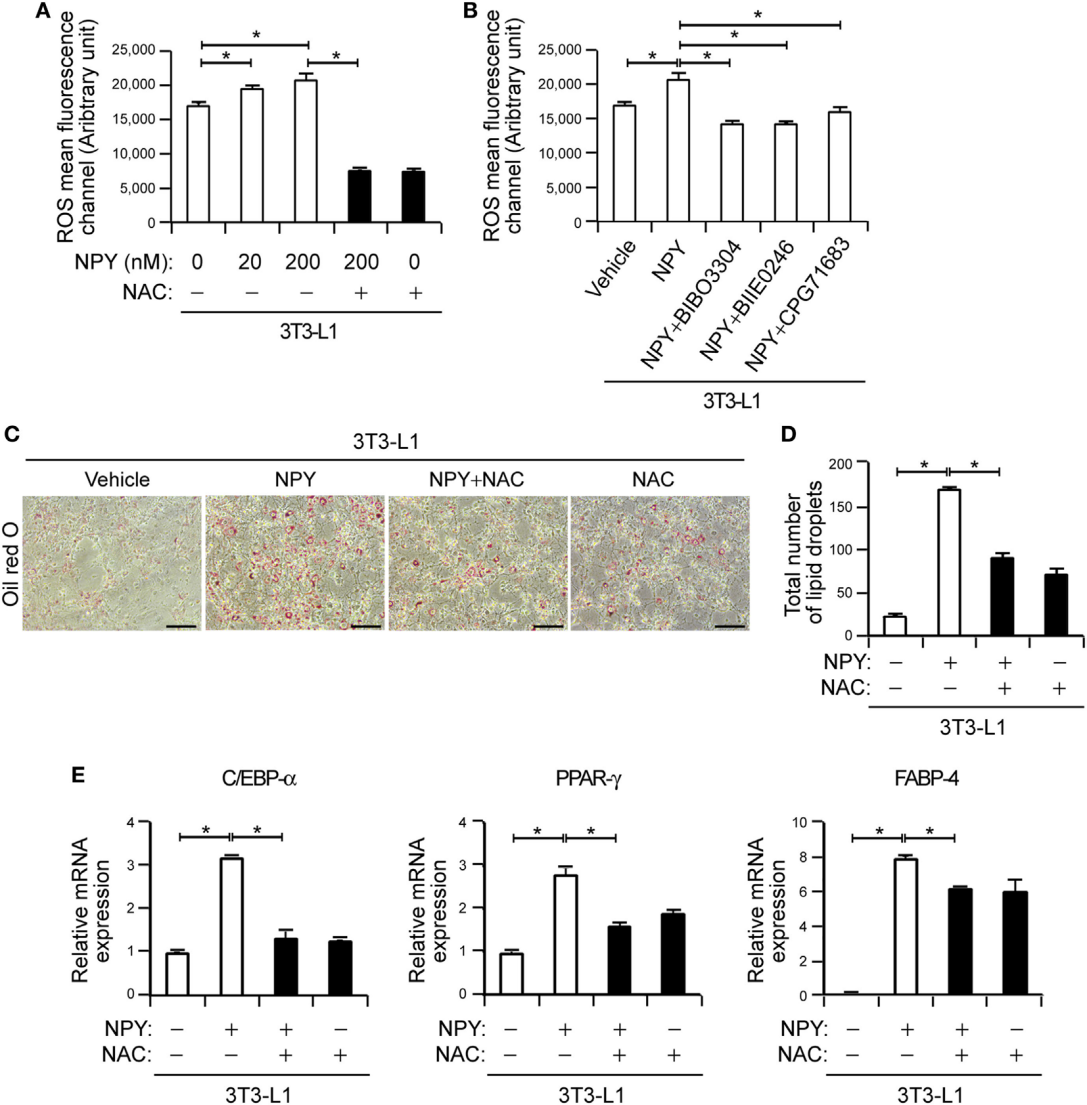
Neuropeptide Y (NPY) induces adipogenesis *via* reactive oxygen species (ROS) production in murine preadipocytes. **(A)** ROS levels were increased in NPY-treated 3T3-L1 cells. The cells were incubated with 2′,7′-dichlorofluorescin diacetate (10 µM) for 30 min at 37°C and subsequently treated with the indicated concentrations of NPY with or without *N*-acetylcysteine (NAC, 10 mM) for 4 h. ROS levels were assessed using a FACS Canto flow cytometry system (*n* = 3). **(B)** The pharmacological inhibition of NPYR blocks NPY-induced ROS production. 3T3-L1 cells were treated with NPY (200 nM) in the presence or absence of NPY1R antagonist (BIBO3304), NPY2R antagonist (BIIE0246), or NPY5R antagonist (CGP71683 hydrochloride) and the ROS levels were subsequently analyzed. *p < 0.05 compared with the vehicle control (*n* = 3). **(C)** 3T3-L1 cells treated with NPY with or without NAC (10 mM) were stained with Oil-Red-O and imaged under a microscope. Scale bar: 100 microm (µM). **(D)** The total number of lipid droplets in **(C)** was quantified (*n* = 5). **(E)** Relative mRNA levels of adipocyte differentiation markers were analyzed using quantitative real-time PCR (qPCR). qPCR data are the fold-changes in the mean vehicle control value (*n* = 4). Data are representative of three independent experiments. *p < 0.05 compared with the vehicle control. *p* values were calculated using Kruskal–Wallis test. The data values represent the mean ± SD.

**Figure 4:**
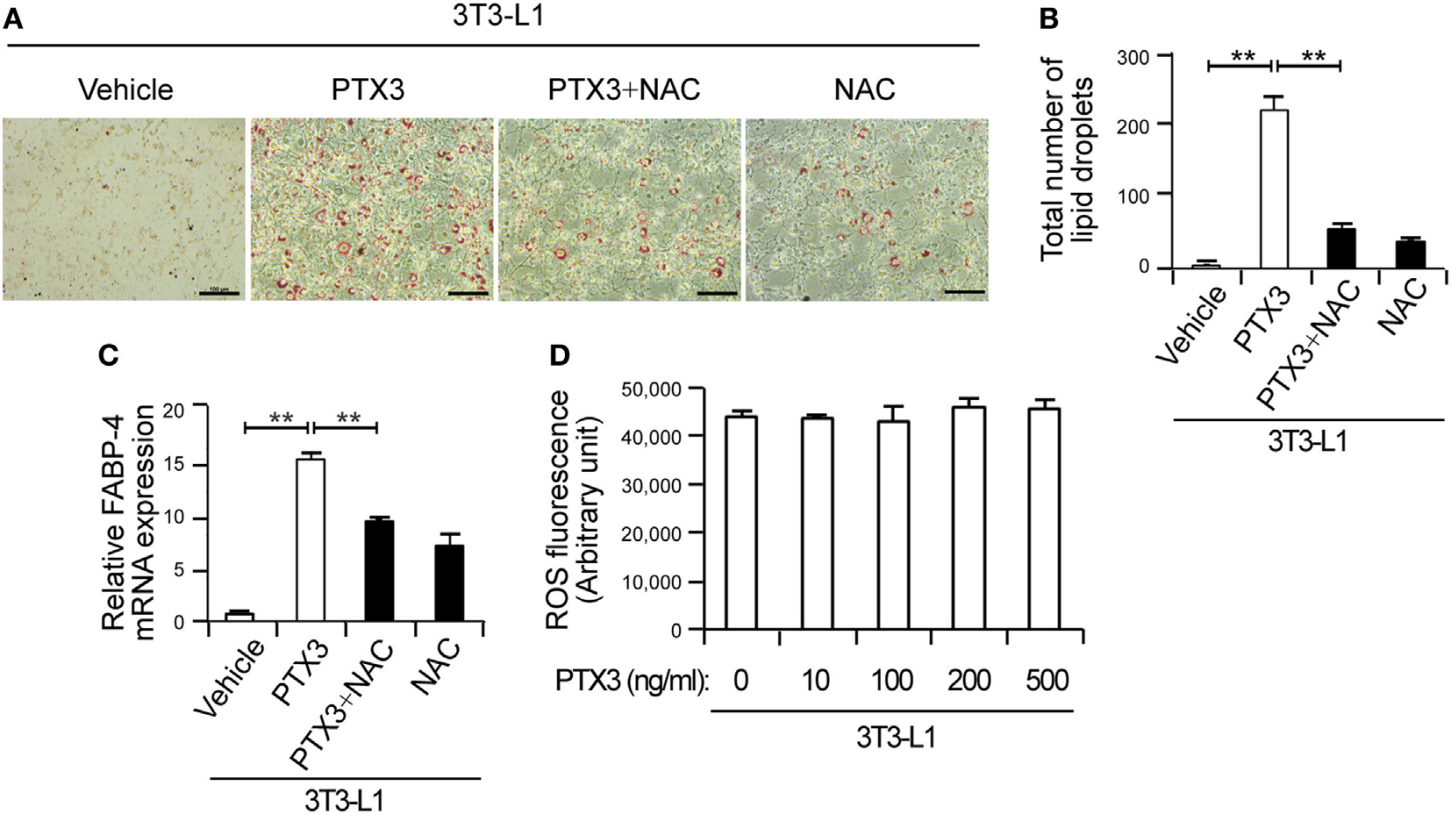
Pentraxin 3 (PTX3)-mediated adipogenesis is inhibited by antioxidants. **(A)** Antioxidants prevent PTX3-mediated increases in lipid accumulation in 3T3 cells. Oil-Red-O staining of 3T3 cells treated with PTX3 with or without the antioxidant *N*-acetylcysteine (NAC) (10 mM) is shown. Scale bar: 100 microm (µM). **(B)** Quantification of the total number of lipid droplets in **(A)** (*n* = 5). **(C)** NAC inhibits PTX3-mediated increases in fatty acid binding protein-4 (FABP-4) expression. FABP-4 expression was analyzed using quantitative real-time PCR (*n* = 4). **(D)** PTX3 has no effect on reactive oxygen species (ROS) production in 3T3-L1 cells. The cells were stained with 2′, 7′-dichlorofluorescin diacetate for 30 min at 37°C and subsequently treated with the indicated concentrations of PTX3 for 4 h. ROS levels were detected using a fluorescent plate reader (*n* = 3). Data are representative of three independent experiments. **p < 0.005 compared with the vehicle control. *p* values were calculated using the Kruskal–Wallis test. Data values represent the mean ± SD.

**Figure 5:**
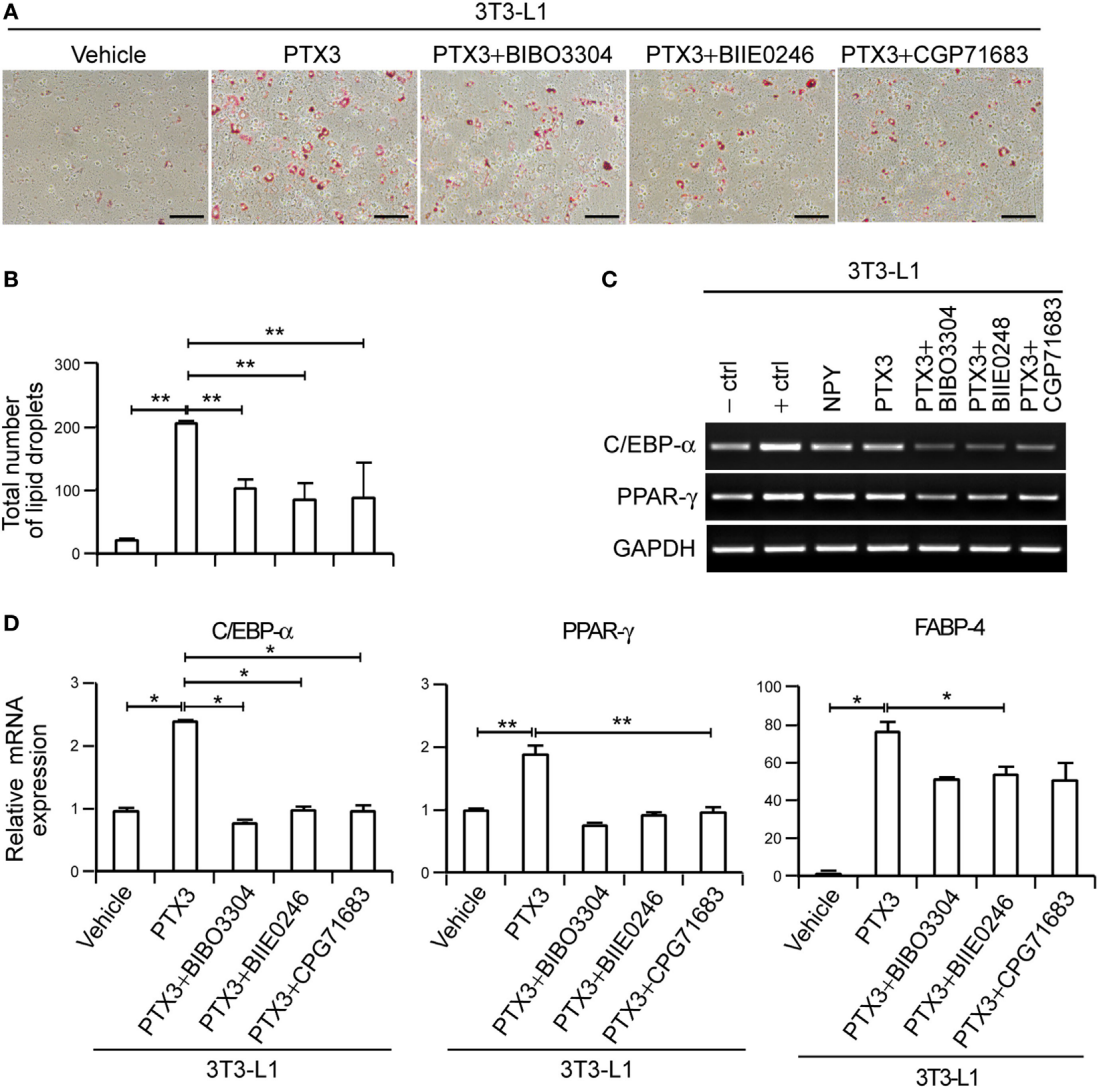
Pentraxin 3 (PTX3)-induced adipogenesis is mediated by neuropeptide Y/NPYR signaling in murine preadipocytes. **(A)** 3T3-L1 preadipocytes were cultured in medium supplemented with a differentiation cocktail for 2 days and then treated with PTX3 (200 ng/ml) with or without NPY1R antagonist (BIBO3304), NPY2R antagonist (BIIE0246), or NPY5R antagonist (CGP71683 hydrochloride) for an additional 4 days without insulin. The cells were fixed, stained with Oil-Red-O, and imaged under a microscope. Scale bar: 100 microm (µM). **(B)** Quantification of the total number of lipid droplets in **(A)** (*n* = 5). **(C)** PTX3 modulates the expression of adipogenesis-associated transcription factors in murine preadipocytes. Adipocytes were treated with PTX3 (200 ng/ml) with or without NPY1R antagonist (BIBO3304) (1 µM), NPY2R antagonist (BIIE0246) (1 µM), or NPY5R antagonist (CGP71683 hydrochloride) (1 µM). Total RNA was isolated from 3T3-L1 preadipocytes, and C/EBP-α and peroxisome proliferator-activated receptor γ (PPAR-γ) levels were determined by RT-PCR. **(D)** PTX3 upregulates the expression of adipocyte differentiation markers *via* NPYR signaling. C/EBP-α, PPAR-γ, and fatty acid binding protein-4 expression levels in cells from **(A)** were determined using quantitative real-time PCR (qPCR). The qPCR data are the fold-changes of the mean vehicle control value (*n* = 4). Data are representative of more than two independent experiments. *p < 0.05 and **p < 0.005 compared with the vehicle control. *p* values were calculated using Kruskal–Wallis test. Data values represent the mean ± SD.

**Figure 6:**
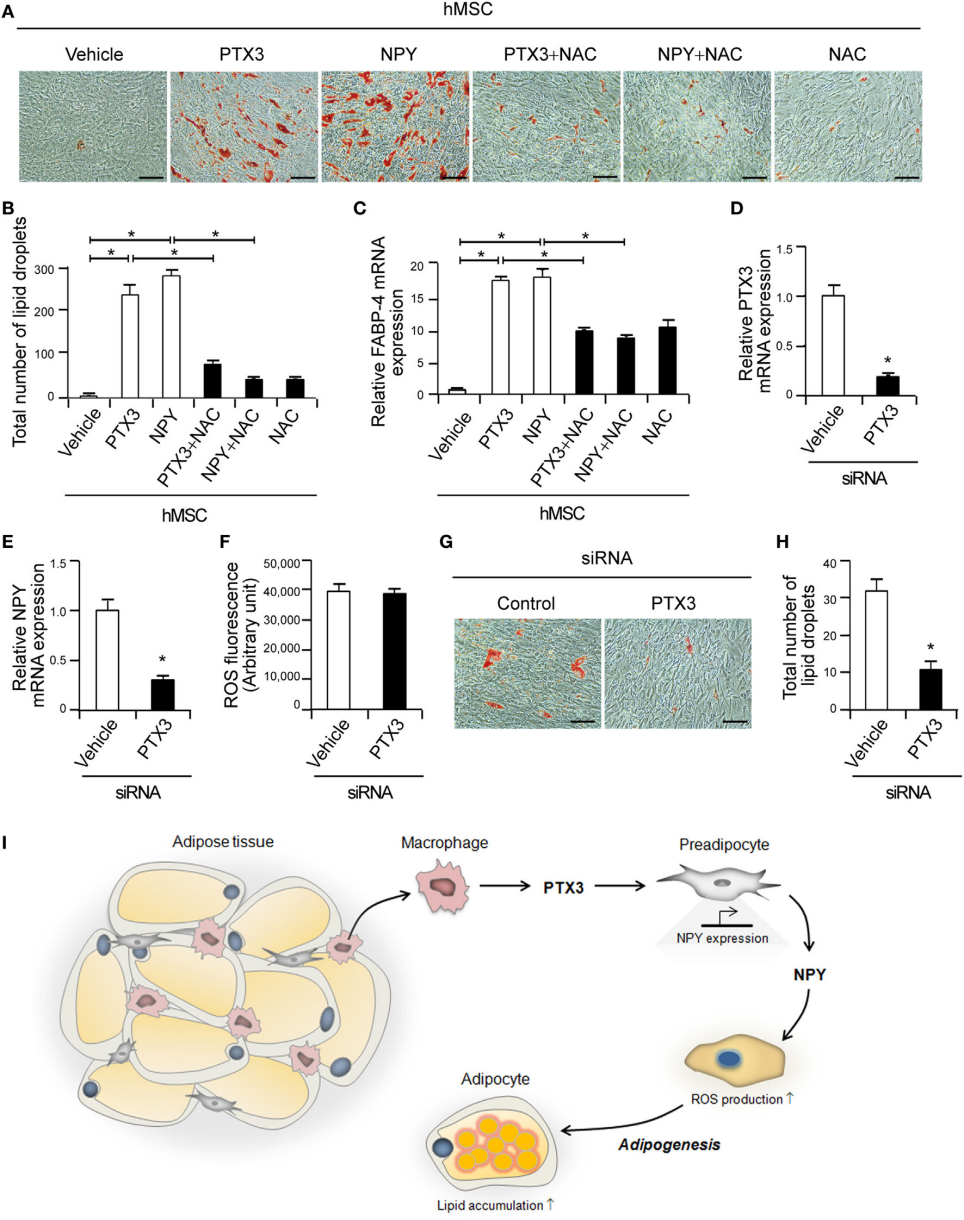
Pentraxin 3 (PTX3) and neuropeptide Y (NPY) induce adipogenesis in human mesenchymal stem cells (hMSCs). **(A)** Antioxidants prevent PTX3- or NPY-mediated increases in lipid accumulation in hMSCs. Oil-Red-O staining of hMSC cells treated with PTX3 or NPY with or without *N*-acetylcysteine (NAC) (10 mM) is shown. Scale bar: 100 microm (µM). **(B)** Quantification of the total number of lipid droplets in **(A)** (*n* = 5). **(C)** The antioxidant NAC inhibits PTX3- and NPY-mediated increases in fatty acid binding protein-4 expression, as determined by quantitative real-time PCR (qPCR) (*n* = 4). **(D)** hMSCs were transfected with either control or PTX3 targeting small interfering RNA (siRNA) and the silencing efficacy of PTX3 targeting siRNA was confirmed with qPCR (*n* = 4). **(E)** NPY mRNA expression level in the cells shown in **(D)** was determined using qPCR (*n* = 4). **(F)** Reactive oxygen species (ROS) levels were analyzed in hMSCs transfected with either control or PTX3 targeting siRNA (*n* = 4). **(G)** Oil-Red-O staining of hMSCs transfected with siRNAs is shown. Scale bar: 100 microm (µM). **(H)** The total number of lipid droplets in **(G)** was quantified (*n* = 5). Data are representative of more than two independent experiments. *p < 0.05 compared with the vehicle control. *p* values were calculated using Kruskal–Wallis test **(B,C)** or Mann–Whitney test **(D–F,H)**. Data values represent the mean ± SD. **(I)** Proposed mechanism of PTX3-induced adipogenesis. PTX3 promotes NPY expression and in turn NPY induces ROS production in adipocytes and adipogenesis. Consequently, PTX3 enhances adipocyte differentiation and subsequent lipid accumulation in adipocytes *via* NPY/NPYR signaling.

All quantitative experiments were performed at least in triplicate and the data values are presented as the mean ± SD. Mann–Whitney test (Figures 1B,C,F and 6D–F,H) or Kruskal–Wallis test (Figures 1E, 2–5 and 6B,C) were used to determine significance. A *p* value of less than 0.05 was considered statistically significant.

## Results

### The Local Elevation of PTX3 Is Involved in Adipogenesis by Inducing the Expression of Adipogenesis-Associated Transcription Factors in Preadipocytes

Previous studies have reported that PTX3 transcripts are elevated in the epididymal fat pads of obese mice (25) and in the visceral adipose tissue of obese human subjects (26, 27). IHC analysis in our present study revealed that the adipocytes of obese db/db mice were enlarged and that PTX3 expression was highly elevated in the adipose tissue from these animals compared to the WT controls (Figures 1A,B) However, the plasma PTX3 level was comparable between WT and db/db mice (Figure 1C), suggesting that PTX3 was elevated locally in the obese adipose tissue.

Adipose tissue expands through adipogenesis, i.e., the differentiation of preadipocytes into the mature adipocytes that store fatty acids (36). Given its elevated expression in the adipose tissue of db/db mice, we reasoned that PTX3 may have effects on adipocyte differentiation and investigated whether it induces lipid accumulation in 3T3-L1 cells, which are murine preadipocytes. Adipocyte differentiation was confirmed by positive Oil-Red-O staining within 6 days of culture in differentiation medium. PTX3 was indeed found to induce the formation of lipid droplets compared with vehicle-treated cells (Figures 1D,E; *p* < 0.0005), suggesting that it effectively induces lipid accumulation in 3T3-L1 cells. Adipocyte differentiation is mediated by the coordinated expression of multiple transcription factors such as C/EBP-α and PPAR-γ (13, 31). We found by qPCR that PTX3 significantly enhanced the expression of C/EBP-α and PPAR-γ in 3T3-L1 cells (Figure 1F, *p* < 0.05). In addition, FABP-4 expression levels were also found to be upregulated in PTX3-treated cells compared with the control cells (Figure 1F, *p* < 0.05). Taken together, our current data suggested that locally elevated PTX3 in the adipose tissue may be involved in the differentiation of murine preadipocytes.

### PTX3 Promotes NPY Expression in Preadipocytes

It was previously reported that NPY expression is induced by inflammatory signals and that this protein participates in obesity-induced adipose tissue inflammation (37). We, therefore, investigated in our current experiments whether pro-inflammatory PTX3 promotes NPY expression in murine preadipocytes. We found that PTX3 but not TNF-α, IL-1β, or IL-8 markedly increased NPY mRNA expression in 3T3 cells (Figure 2A) but not in bone marrow-derived macrophages (Figure 2B). This induction of NPY mRNA expression by PTX3 was up to sixfold at 10 ng/ml and was sustained at an elevated level at 100 and 200 ng/ml doses (Figures 2C,D). RT-PCR and qPCR assays revealed that PTX3 treatment markedly upregulated NPY2R and NPY5R mRNA expression in 3T3-L1 cells (Figures 2C,D, *p* < 0.05), but did not affect NPY1R expression (Figures 2C,D). These results indicated that PTX3 induces the expression of NPY and its receptors in murine preadipocytes.

### NPY Induces Adipogenesis *via* ROS Production in Murine Preadipocytes

Previous studies have demonstrated that NPY induces lipid synthesis in the 3T3-L1 cell line (13, 31, 36) and that ROS promote adipogenesis by enhancing the activation of adipogenic transcription factors (38). We confirmed that NPY also stimulates ROS production, which is abolished by the general antioxidant NAC (39) (Figure 3A, *p* < 0.05). Interestingly, antagonists of NPY1R, NPY2R, and NPY5R also significantly inhibited NPY-mediated ROS production (Figure 3B, *p* < 0.05), indicating that NPY stimulates ROS production in preadipocytes by binding to NPY receptors.

To further investigate the role of NPY-mediated ROS production in adipocyte differentiation, we evaluated the effects of NAC on this process. Oil-Red-O staining demonstrated that NPY treatment promotes lipid accumulation (Figures 3C,D, *p* < 0.05), consistent with previous findings (13, 31). Moreover, NPY-mediated adipogenic differentiation was inhibited in 3T3-L1 cells when cultured in differentiation medium supplemented with NAC (Figures 3C,D, *p* < 0.05), suggesting that ROS production contributes to adipogenic differentiation in these cells. Consistent with previous reports (13, 31), we further found in our current analysis that NPY induced a 3.20 ± 0.01-, 2.75 ± 0.22-, and 7.90 ± 0.02-fold increase in the expression levels of C/EBP-α, PPAR-γ, and FABP-4, respectively (Figure 3E, *p* < 0.05). This NPY-induced expression of adipocyte differentiation markers was diminished by NAC (Figure 3E, *p* < 0.05). Taken together, our current results suggest that NPY induces adipogenesis by enhancing ROS production in murine preadipocytes.

### PTX3 Mediates Adipogenesis *via* NPY-Dependent ROS Production in Preadipocytes

Given our observations of PTX3-mediated adipogenesis and PTX3-induced NPY expression, we reasoned that PTX3 may enhance adipocyte differentiation in a NPY-induced ROS-dependent manner. NAC nearly abolished PTX3-mediated adipogenic differentiation in 3T3-cells (Figures 4A,B) and also reduced the expression of FABP-4, a marker of terminal adipogenic differentiation (Figure 4C), raising the possibility that ROS production may contribute to PTX3-mediated adipogenic differentiation in these cells. However, exogenous PTX3 failed to directly induce ROS production in 3T3-L1 cells (Figure 4D).

We speculated that PTX3 might trigger lipid accumulation *via* NPY signaling and indeed found that NPY receptor antagonists inhibited the stimulatory effect of PTX3 on lipid synthesis. NPY1R antagonist (BIBO3304), NPY2R antagonist (BIIE0246), and NPY5R antagonist (CGP71683 hydrochloride) elicited a marked 2.02 ± 0.26-, 2.55 ± 0.79-, and 2.94 ± 0.89-fold reduction, respectively, of PTX3-induced lipid accumulation (Figures 5A,B). Furthermore, the anti-adipogenic effects of these NPYR antagonists was associated with lower adipogenesis-associated transcription factor levels including C/EBP-α, PPAR-γ, and FABP-4 (Figures 5C,D, *p* < 0.05), suggesting that the stimulatory effect of PTX3 on adipogenesis is inhibited by NPY receptor antagonists. Our findings thus indicated that PTX3 promotes adipogenesis not by directly inducing ROS but by increasing NPY-dependent ROS production.

### PTX3 and NPY Promote Adipogenic Differentiation From Human Mesenchymal Stem Cells

hMSCs are capable of differentiating into osteogenic, chondrogenic, and adipogenic lineages (32). We induced hMSC differentiation into adipocytes to determine the effects of PTX3 and NPY on adipogenesis in other cell types. Adipocyte differentiation was confirmed by positive Oil-Red-O staining within 9 days of culture in differentiation medium (Figure 6A). Similar to our observations in 3T3-L1 cells, PTX3 and NPY significantly enhanced lipid accumulation in hMSCs (Figures 6A,B, *p* < 0.05). Moreover, PTX3- and NPY-mediated adipogenic differentiation was also nearly abolished in hMSCs cultured in differentiation medium supplemented with NAC (Figures 6A,B, *p* < 0.05), suggesting that ROS production contributes to adipogenic differentiation in hMSCs. As found in 3T3-L1 cells, PTX3 and NPY significantly upregulated the expression of the adipogenesis differentiation marker, FABP-4, an effect, which was inhibited by the antioxidant NAC (Figure 6C, *p* < 0.05). This confirmed the promoting effects of PTX3 and NPY on adipocyte differentiation and lipid accumulation.

To confirm the involvement of PTX3 in adipogenesis, endogenous PTX3 was silenced in hMSCs using siRNAs targeting PTX3. The expression of PTX3 mRNA was successfully reduced to approximately 19% of the level in hMSCs transfected with control siRNA (Figure 6D). We examined the effect of PTX3 silencing on NPY expression and ROS production and found that transfection of PTX3 siRNA decreased NPY mRNA expression (Figure 6E) but had no significant effect on ROS production in hMSCs (Figure 6F). More importantly, PTX3-silenced cells showed reduced lipid accumulation compared to those transfected with control siRNA (Figures 6G,H), suggesting that PTX3 is involved in adipogenesis.

## Discussion

Adipose tissue exhibits an elevated expression of proinflammatory cytokines in obese subjects, including TNF-α and IL-6 (40, 41), which then promotes fatty acid release into the bloodstream *via* the induction of adipocyte lipolysis (42). Adipose tissue macrophages are responsible for a significant proportion of the TNF-α and IL-6 expression in the body (14) and TNF-α induces PTX3 expression in various cells including adipocytes (24, 25). This raised the possibility that PTX3 is involved in adipogenesis, which we have now confirmed in our current analysis. Several earlier reports had demonstrated that PTX3 expression is locally elevated in the adipose tissue of obese patients (26, 27). In our current study, we confirmed that the PTX3 expression was locally elevated in obese adipose tissue (Figure 1). Moreover, PTX3 enhanced NPY expression from adipocytes (Figure 2) and promoted adipocyte differentiation *via* NPY/NPYR signaling in 3T3-L1 cells (Figure 5). These results have revealed a new and distinct adipogenic role of PTX3 as our present report is the first to identify the promoting effects of PTX3 in adipogenesis.

It is well established that NPY is produced from adipocytes in subcutaneous and VATs as well as by the sympathetic nervous system (43). Both central nervous system- and adipose tissue-derived NPY regulates angiogenesis, vasoconstriction, fertility, and hormone secretion (43). NPY directly induces the formation and growth of adipose tissue by promoting macrophage infiltration and stimulating preadipocyte proliferation and differentiation (36, 44). Indeed, a previous study has reported that experimental obesity model exhibited an elevated activity of NPY and its receptors (45). NPY promotes preadipocyte proliferation and adipogenesis in human white adipose tissue (31, 36, 44). Moreover, NPY raises the risk of obesity by upregulating NPY and NPY2R expression in the abdominal fat and its administration in rodents leads to lipogenic enzyme activation in adipose tissues (36), demonstrating its critical role in adipogenesis. The signaling that induces NPY/NPYR may also participate in the regulation of adipogenesis. Our present results have indicated that PTX3 induces the expression of NPY, NPY2R, and NPY5R (Figure 2), suggesting a promoting effect upon adipocyte differentiation through NPY signaling.

Previous studies have reported that ROS are generated during the process of adipogenesis (46) and that the adipose tissue in obese mice exhibits elevated ROS levels (47). Lee et al. have demonstrated that ROS are critical for mitotic clonal expansion of preadipocytes, ultimately accelerating adipocyte differentiation (38). In our present analysis, we observed that NPY expression was elevated in adipose tissue from the db/db mouse compared with the control animals (data not shown) and that NPY induces ROS production in adipocytes (Figure 3). We here found that NPYR antagonists, as well as general antioxidants, could diminish NPY-dependent ROS production and in turn block NPY-mediated adipocyte differentiation (Figure 3), reinforcing the definite promoting effect of NPY on ROS production leading to accelerated adipogenesis. However, further studies will be required to determine the precise mechanisms underlying the NPY-mediated induction of ROS in adipocytes.

Previous findings have suggested that NPY induces lipid synthesis in the murine preadipocyte cell line 3T3-L1 (13, 31) by activating NPY1R, NPY2R, or NPY5R (31, 36, 44). We here demonstrated that antagonists of NPY1R, NPY2R, or NPY5R inhibited PTX3-mediated lipid accumulation and PTX3-induced expression of adipogenesis transcription factors (Figure 5). This lends further supporting evidence for the role of PTX3 in the induction of adipogenesis *via* NPY signaling. NPYR in adipocytes appears to play an important role in PTX3- and NPY-induced stimulation of adipogenesis, implying that NPYRs might be a potential therapeutic target for the treatment of obesity. Kuo et al. reported that pharmacological and genetic inactivation of NPY2R led to a reduction in adipogenesis and abdominal obesity, providing support for NPY-NPYR-based drugs as candidate anti-obesity-therapeutics (36). Indeed, a variety of antagonists of NPYR have now been tested as anti-obesity drugs (48, 49).

In conclusion, our present study, to the best of our knowledge, provides the first reported evidence of (1) PTX3-induced NPY expression in adipocytes, (2) PTX3-mediated induction of adipocyte differentiation *via* NPY/NPYR signaling, and (3) NPY-induced ROS production in adipocytes (Figure 6I). These findings provide important new insights into the lipogenic role of PTX3 in adipogenesis as this will inform the development of new therapies for the treatment and/or prevention of obesity.

## Ethics Statement

Human mesenchymal stem cells (hMSCs) were isolated from human fat tissue in accordance with Institutional Review Board guidelines (IRB number 2012-0283, Asan Medical Center, Korea). The protocol was approved by the Institutional Review Board guidelines (Asan Medical Center, Korea). All subjects gave written informed consent in accordance with the Declaration of Helsinki.

## Author Contributions

M-KS, BC, E-YK, and J-EP performed experiments; EH, HL, MK, J-EK, SK, and E-JC designed the study; M-KS, BC, and E-JC prepared the manuscript.

## Conflict of Interest Statement

The authors declare that the research was conducted in the absence of any commercial or financial relationships that could be construed as a potential conflict of interest.

## Abbreviations

C/EBP-α, CCAAT-enhancer-binding protein-α; FABP-4, fatty acid binding protein-4; IBMX, 3-isobutyl-1-methylxanthine; IL-1β, interleukin-1β; MSC, mesenchymal stem cell; NAC, *N*-acetylcysteine; NPY, neuropeptide Y; NPYR, neuropeptide Y receptor; PPAR-γ, peroxisome proliferator-activated receptor γ; PTX3, pentraxin 3; qPCR, quantitative real-time PCR; ROS, reactive oxygen species; TNF-α, tumor necrosis factor-α; WT, wild type.
