# Supplementary material for: Elevated Pentraxin 3 in Obese Adipose Tissue Promotes Adipogenic Differentiation by Activating Neuropeptide Y Signaling
Source: Front Immunol. 2018 Jul 30;9:1790. doi: 10.3389/fimmu.2018.01790 (PMC6077621; doi:10.3389/fimmu.2018.01790)
Supplement: Supplementary file 1 [file Table_1.DOC]

**Supplemental Information**

Supplementary Table I. PCR primer sequences

| Gene name | Primer sequence | |
| --- | --- | --- |
| murine GAPDH | Forward | 5′-TGGCCTTCCGTGTTCCTAC-3′ |
|  | Reverse | 5′-GAGTTGCTGTTGAAGTCGCA-3′ |
| murine NPY | Forward | 5′-ATGCTAGGTAACAAGCGAATGG-3′ |
|  | Reverse | 5′-TGTCGCAGAGCGGAGTAGTAT-3′ |
| murine NPY1R | Forward | 5′-TGATCTCCACCTGCGTCAAC-3′ |
|  | Reverse | 5′-ATGGCTATGGTCTCGTAGTCAT-3′ |
| murine NPY2R | Forward | 5′-GCCAGGGCACACTACTCCTA-3′ |
|  | Reverse | 5′-CTACCCCTAGCAAGATGATGGA-3′ |
| murine NPY5R | Forward | 5′-TTTGTCACGGAGAACAATACTGC-3′ |
|  | Reverse | 5′-TGCGCTTTTTCATAACAGCCAT-3′ |
| murine PTX3 | Forward | 5′-TTTTGGAAGCGTGCATCCTGT-3′ |
|  | Revers | 5′-CACCACCAACACTAGGGACTG-3′ |
| murine C/EBP-α | Forward | 5′-CAAGAACAGCAACGAGTACCG-3′ |
|  | Revers | 5′-GTCACTGGTCAACTCCAGCAC-3′ |
| murine PPAR-γ | Forward | 5′-TTTTCCGAAGAACCATCCGATT-3′ |
|  | Reverse | 5′-ATGGCATTGTGAGACATCCCC-3′ |
| murine FABP-4 | Forward | 5′-AAGGTGAAGAGCATCATAACCCT-3′ |
|  | Reverse | 5′-TCACGCCTTTCATAACACATTCC-3′ |
| human GAPDH | Forward | 5′TGTTGCCATCAATGACCCCTT-3′ |
|  | Reverse | 5′-CTCCACGACGTACTCAGCG-3′ |
| human FABP-4 | Forward | 5′-ACTGGGCCAGGAATTTGACG-3′ |
|  | Reverse | 5′-CTCGTGGAAGTGACGCCTT-3′ |
